# The Epitopic and Structural Characterization *of Brucella suis* Biovar 2 O-Polysaccharide Demonstrates the Existence of a New M-Negative C-Negative Smooth *Brucella* Serovar

**DOI:** 10.1371/journal.pone.0053941

**Published:** 2013-01-15

**Authors:** Mona V. Zaccheus, Tara Ali, Axel Cloeckaert, Michel S. Zygmunt, Andrej Weintraub, Maite Iriarte, Ignacio Moriyón, Göran Widmalm

**Affiliations:** 1 Department of Organic Chemistry, Arrhenius Laboratory, Stockholm University, Stockholm, Sweden; 2 Karolinska Institute, Department of Laboratory Medicine, Division of Clinical Microbiology, Karolinska University Hospital, Stockholm, Sweden; 3 INRA, UMR1282 Infectiologie et Santé Publique, Nouzilly, France; 4 Université François Rabelais de Tours, UMR1282 Infectiologie et Santé Publique, Tours, France; 5 Institute of Tropical Health and Department of Microbiology and Parasitology, University of Navarra, Pamplona, Spain; University of Helsinki, Finland

## Abstract

The brucellae are Gram-negative bacteria that cause an important zoonosis. Studies with the main *Brucella* species have shown that the O-antigens of the *Brucella* smooth lipopolysaccharide are α-(1→2) and α-(1→3)-linked *N*-formyl-perosamine polysaccharides that carry M, A and C (A = M, A>M and A<M) epitopes relevant in serodiagnosis and typing. We report that, in contrast to the *B. suis* biovar 1 O-antigen used as a reference or to all described *Brucella* O-antigens, *B. suis* biovar 2 O-antigen failed to bind monoclonal antibodies of C (A = M), C (M>A) and M specificities. However, the biovar 2 O-antigen bound monoclonal antibodies to the *Brucella* A epitope, and to the C/Y epitope shared by brucellae and *Yersinia enterocolitica* O:9, a bacterium that carries an *N*-formyl-perosamine O-antigen in exclusively α-(1→2)-linkages. By ^13^C NMR spectroscopy, *B. suis* biovar 1 but not *B. suis* biovar 2 or *Y. enterocolitica* O:9 polysaccharide showed the signal characteristic of α-(1→3)-linked *N*-formyl-perosamine, indicating that biovar 2 may altogether lack this linkage. Taken together, the NMR spectroscopy and monoclonal antibody analyses strongly suggest a role for α-(1→3)-linked *N*-formyl-perosamine in the C (A = M) and C (M>A) epitopes. Moreover, they indicate that *B. suis* biovar 2 O-antigen lacks some lipopolysaccharide epitopes previously thought to be present in all smooth brucellae, thus representing a new brucella serovar that is M-negative, C-negative. Serologically and structurally this new serovar is more similar to *Y. enterocolitica* O:9 than to other brucellae.

## Introduction

Brucellosis is one of the major bacterial zoonoses in the world [1]. This disease is caused by members of the genus *Brucella,* a group of Gram-negative microorganisms that behave as facultative intracellular parasites of a large variety of mammals. Infected livestock is the source of human brucellosis, a grave and debilitating disease that requires prolonged antibiotic treatment. The genus *Brucella* includes several species, three of which (*B. abortus*, *B. melitensis* and *B. suis*) account for the vast majority of infections in domestic livestock and humans. These are further divided into biovars for epidemiological purposes. Regardless of the biovar, the main hosts of *B. abortus* and *B. melitensis* are cattle and small ruminants, respectively, and genetic analyses show that the strains of these two species form two distinct clusters within the genus [2]. On the other hand, the strains presently grouped under *B. suis* do not cluster together and are usually found in swine (biovars 1, 2 and 3), wild boars and hares (biovar 2), reindeer (biovar 4), and wild rodents (biovar 5) [2]–[4]. Because of their zoonotic nature and importance in ruminant husbandry and in the dairy industry, research on *Brucella* virulence, physiology and antigenic structure has been carried out mostly on *B. abortus* and *B. melitensis*. *B. suis* has received comparatively meager attention and, despite the greater internal diversity and wide host range, research has been focused on biovar 1.

*B. abortus*, *B. melitensis* and *B. suis* cells carry a smooth (S) lipopolysaccharide (LPS), a surface molecule that is a major virulence factor and the most important serodiagnostic antigen. The O-polysaccharide (or O-antigen) section of this S-LPS is a homopolymer of *N-*formyl-perosamine (d-Rha*p*4NFo) in α-(1→2)- and α-(1→3)-linkages [5] that is bound to the lipid A through a core oligosaccharide. From extensive genetic studies in *B. abortus* biovar 1 and *B. melitensis* biovar 1, O-antigen polymerization requires at least four glycosyltransferase genes (*wboA, wboB, wbkA and wbkE*) [6]. All O-antigens of S *Brucella* studied by NMR so far (*B. melitensis* 1 and 3, *B. abortus* biovar 1 and *B. suis* biovar 4) contain at least one α-(1→3)-linked d-Rha*p*4NFo residue, with *B. melitensis* biovar 1 showing the highest proportion (four contiguous α-(1→2)-linked sugars followed by one α-(1→3)-linkage) [7]. As demonstrated with monoclonal antibodies (MAb), these linkages create three basic epitopes: A (five or more contiguous α-(1→2)-linked d-Rha*p*4NFo units), M (which strictly requires α-(1→3)-linked sugars), and C (which includes α-(1→2)-linked tri- or tetrasaccharides) [7]–[10]. However, as expected from the polysaccharide structures and the variability in affinity intrinsic to antibody binding sites, these three basic epitopes in *Brucella* O-polysaccharide are not discrete entities and behave as overlapping epitopes. In fact, based on the relative MAb binding in enzyme-linked immunosorbent assays (ELISA) to A- and M-dominant *Brucella* strains and to *Yersinia enterocolitica* O:9 (which carries a d-Rha*p*4NFo homopolymer having only α-(1→2)-linkages [11]), the C epitope has been subdivided into five subsets: C (M>A), C (A = M), C/Y (M>A), C/Y (A = M), and C/Y (A>M), where C indicates specificity for *Brucella* and for C/Y *Brucella*-*Y*. *enterocolitica* O:9 cross-reactivity [12], [13]. The A and M epitopes are not uniformly distributed in the genus: *B. abortus* biovars 1, 2, 3 and 6 as well as *B. suis* 1, 2 and 3 carry the A epitopes but not the M, while *B. melitensis* biovar 1, *B. abortus* biovars 4, 5 and 9 and *B. suis* biovar 5 have M but not A epitopes [14].

It has been reported that *B. suis* biovar 2 fails to react with MAb specific for the C (A = M) and C (M>A) epitopes [15] suggesting unknown structural peculiarities. *B. suis* biovar 2 represents an emerging disease in domestic swine throughout Europe and it has the striking feature of not being overtly virulent for humans [16], [17], [18]. Because of the importance of S-LPS in the biology of brucellae, elucidation of the fine structure of *B. suis* biovar 2 O-antigen should help both to understand the nature of the C and C/Y epitopes and to better characterize this atypical and increasingly important biovar. Here, we confirm a different epitopic structure of the O-antigen of *B. suis* biovar 2 and show that it is closer to that of *Y*. *enterocolitica* O:9 than to those of *B. suis* biovar 1 or other S *Brucella* characterized to date. We could not detect α-(1→3)-linked d-Rha*p*4NFo residues in the *B. suis* 2 O-antigen. This is in contrast to the O-antigen of other S brucellae studied thus far and strongly suggests a role for α-(1→3)-linked d-Rha*p*4NFo in the C (A = M) and C (M>A) epitopes.

## Results and Discussion

**Figure 1:**
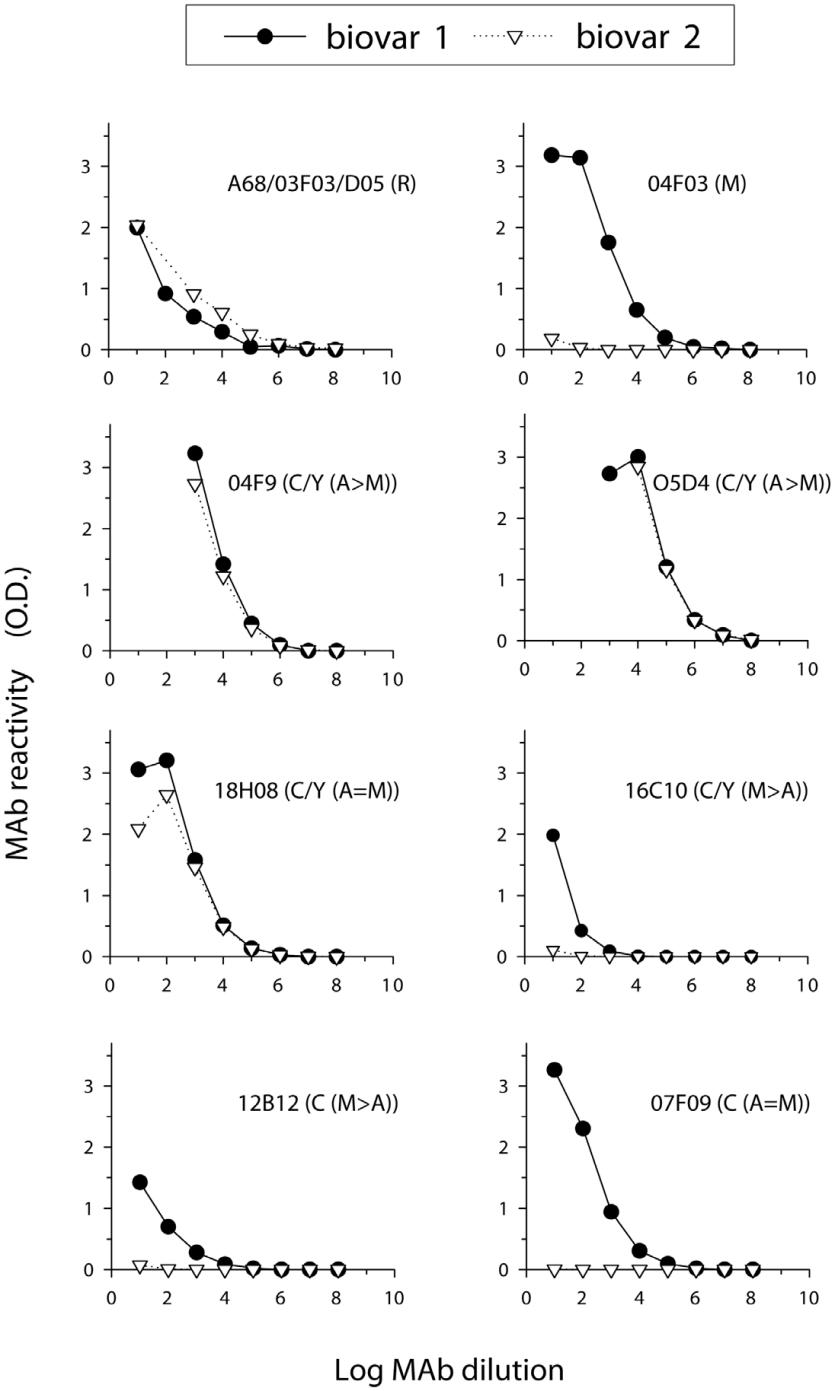
Reactivity of MAb of various LPS specificities with *B. suis* biovar 1 and biovar 2 PS. The logarithm is to the base 2.

To determine the epitopic structure of the *B. suis* biovar 2 O-antigen, we examined the polysaccharide (PS, i.e. the core-O-antigen polysaccharide) obtained by acid hydrolysis of the corresponding LPS using the PS from the well-characterized *B. suis* biovar 1 LPS as a reference [7]. These PS were first analyzed by ELISA with a panel of MAb specific for *Brucella* rough LPS and for the overlapping epitopes described previously in *Brucella* O-antigens (Figure 1 and Table 1). Both PS reacted with the MAb of rough specificity, showing that they contained the core oligosaccharide epitopes that are present in rough LPS. Thus, they were the result of the hydrolysis of the mature S-LPS, and not O-antigen precursors or related *Brucella* polysaccharides of uncertain epitopic structure [9], [19]. When the O-antigen MAb were used, it was observed that MAb 04F9 (C/Y (A>M)), 05D4 (C/Y (A>M)) and 18H08 (C/Y (A = M)) (all detecting the *Y. enterocolitica* O:9-*Brucella* C/Y common epitopes) reacted with the PS of both *B. suis* biovars (Figure 1). Since *Y. enterocolitica* O:9 lacks α-(1→3)-linkages, the reactivities of these MAb (Table 1) indicate that they do not have an absolute requirement for the presence of α-(1→3)-linked residues and that their binding to the M-dominant brucellae can be accounted for by an ability to recognize a short range of α-(1→2)-linked oligomers. Accordingly, for a more detailed analysis of the epitopic structure of *B. suis* biovar 2 PS, we tested MAb showing a restricted or preferential reactivity with the M epitope, which has been related to the existence of α-(1→3)-linkages [7]. The M specific MAb 04F03, while reacting with the PS of *B. suis* biovar 1, failed to bind the biovar 2 PS (Figure 1). Similarly, MAb 16C10, of C/Y (M>A) specificity, failed to bind appreciably to the biovar 2 PS even though it reacted weakly with the biovar 1 PS (Figure 1). Since the latter MAb has been previously defined as M>A to indicate its higher binding to M-dominant O-antigens (Table 1), this result is consistent with the failure of the M-specific MAb 04F03 to bind to *B. suis* biovar 2 but not to biovar 1 PS. Moreover, both MAb 04F03 and MAb 16C10 indicated a significant difference between the PS of biovar 1 and 2 at the level of structure responsible for the M epitope. All these results suggest that the O-antigen of *B. suis* biovar 2: (i), could be serologically indistinguishable from the O-antigen of *Y*. *enterocolitica* O:9, at least with the MAb of the specificities defined so far; and (ii), that it could have levels of α-(1→3)-linkages far below those of other S brucellae described to date.

**Table 1:** Characteristics, origin and reactivity with *B. suis* biovar 1 and 2 PS of the MAb used.^1.^

			Reported reactivity with S-LPS or PS from				Reactivity with PS of	
MAb	Specificity	Isotype	*B. abortus*biovar 1	*B. melitensis*biovar 1	*Y. enterocolitica* O:9	References	*B. suis*biovar 1	*B. suis*biovar 2
A68/03F03/D05	R (LPS core)	IgG2b	N.D.^2^	N.D.	N.D.		+++	+++
04F03	M	IgM	+/−	++++	−	[9], [12], [13]	++++	−
04F9	C/Y (A>M)	IgG2a	++++	+/−	+++	[12], [13], [35], [36]	++++	++++
05D4	C/Y (A>M)	IgG1				This work	++++	++++
18H08	C/Y (A = M)	IgA				[12], [13]	++++	++++
16C10	C/Y (M>A)	IgG3	++	++++	++	[12], [13], [36]	++	−
12B12	C (M>A)	IgG3	++	++++	−	[9], [12], [13]	++	−
07F09	C (A = M)	IgG1	++++	++++	−	[9], [12], [13]	++++	−

1 Reactivity is reported as no reactivity (-) to strong (++++) as judged from the titers reported in the corresponding references.

2 N.D. = not determined.

**Figure 2:**
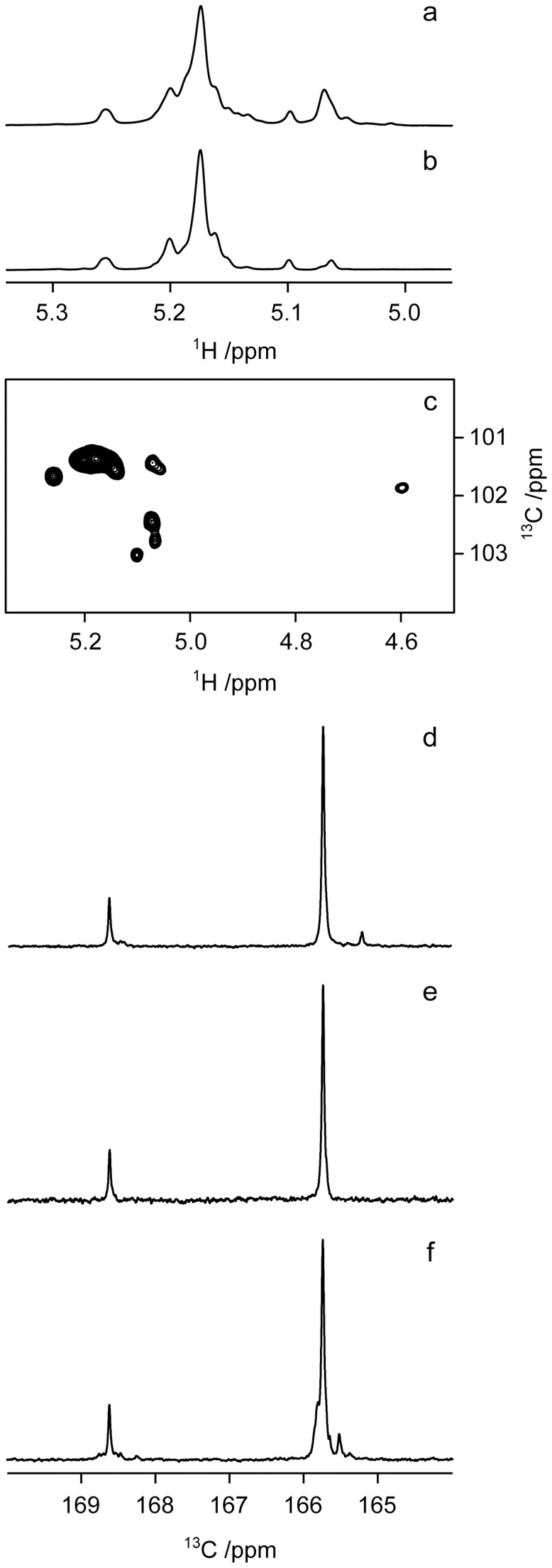
Selected anomeric region of the ^1^H NMR spectrum from *B. suis* biovar 1 PS (a), biovar 2 PS (b); the anomeric region of the ^1^H,^13^C-HSQC NMR spectrum from *B. suis* biovar 1 PS (c); Selected carbonyl region of the ^13^C NMR spectrum from *B. suis* biovar 1 PS with a resonance of low intensity at 165.2 ppm (d), and biovar 2 PS (e); selected carbonyl region of the ^13^C NMR spectrum from *Y*. *enterocolitica* O:9 PS with resonances of low intensity at 165.5 and 165.8 ppm (f). The major ^13^C resonances from *N*-formyl groups are observed at 165.7 and 168.6 ppm.

To test the first hypothesis, we used MAb of C specificity, an epitope typical of S brucellae but absent in *Y*. *enterocolitica* O:9. As can be seen in Figure 1, MAb 12B12 (C (M>A)) and 07F09 (C (A = M)) reacted with the PS of *B. suis* biovar 1 but not with the PS of *B. suis* biovar 2, thus confirming that *B. suis* 2 PS is not distinguishable from its *Y. enterocolitica* counterpart with the MAb specificities defined thus far. To test the second hypothesis, we investigated the presence of α-(1→3)-linkages by NMR spectroscopy. Consistent with the serological results, the anomeric region of the ^1^H NMR spectra revealed differences between the *B. suis* biovar 1 and 2 PS. In particular, the peak at ∼5.07 ppm was more intense in the *B. suis* biovar 1 PS spectrum than from the biovar 2 PS spectrum (Fig. 2a and 2b). This difference is reminiscent of ^1^H NMR spectral changes taking place when the PS of biovar 1 *B. melitensis* 16M and biovar 1 *B. abortus* 1119-3 are mixed [α-(1→3)/α-(1→2) ratio for the mixture c.a. 1∶2] [7]. Thus, the results from analyses of the ^1^H NMR spectra also suggest that the O-antigen from *B. suis* biovar 1 contains a higher amount of α-(1→3)-linkages than that of biovar 2.

To confirm the above spectral analysis, the PS from *B. suis* biovar 1 and 2 were further analyzed by two-dimensional NMR spectroscopy. In the anomeric region of the ^1^H,^13^C-HSQC spectra from the two PS, six cross-peaks in common were observed at δ_H_/δ_C_ 5.26/101.6, 5.20/101.3, 5.17/101.3, 5.10/103.0 5.06/102.7 and 4.60/101.8. Furthermore, four additional cross-peaks were present in the biovar 1 PS at δ_H_/δ_C_ 5.14/101.5, 5.07/102.4, 5.07/101.4 and 5.05/101.5 (Fig. 2c) but not in biovar 2 PS. The distinction between 2- or 3-linked residues may be performed by analysis of ^13^C NMR chemical shifts [20], [21] or the glycosylation shifts, i.e., chemical shift displacements as a result of substitution by a sugar residue [22] at either O2 or O3 of the d-Rha*p*4NFo residues. For a non-substituted α-linked d-Rha*p*4NFo, the ^13^C chemical shifts of C2 and C3 are ∼70 and ∼69 ppm, respectively, whereas glycosylation displace these chemical shifts to ∼78 and ∼77 ppm, respectively. The ^13^C chemical shifts at the glycosylation positions per se may not be sufficient to easily distinguish between the different substitutions but the large glycosylation shifts of ∼8 ppm facilitate their differentiation. The two PS were subsequently analyzed by ^1^H,^13^C-H2BC and ^1^H,^13^C-HMBC experiments [23], [24] in which heteronuclear ^1^H,^13^C-correlations over two bonds are observed in the first experiment and correlations over two and/or three bonds are present in the second one. Correlations over three bonds in ^1^H,^13^C-HMBC spectra may be due to intraresidue as well as interresidue *J*-couplings and are thus not necessarily conclusive with respect to the substitution position in a d-Rha*p*4NFo residue that is part of a polysaccharide. In the ^1^H,^13^C-H2BC spectrum of the *B. suis* biovar 1 PS, but not of biovar 2, correlations from δ_H_ 5.07 were observed to 69.6 and 78.3 ppm. The former correlation supports the presence of an α-linked d-Rha*p*4NFo residue in the PS of biovar 1 that is not 2-substituted; consequently, it should be 3-substituted (unless it is the terminal residue of the polysaccharide).

In the ^1^H,^13^C-HSQC spectra the ^13^C spectral region between 51 and 58 ppm also showed marked differences. Major cross-peaks were observed at δ_H_/δ_C_ 3.99/52.7 and 3.42/57.7. In the PS from both strains, the H2/C2 cross-peak from *N*-acetyl-quinovosamine (QuiNAc) was observed at δ_H_/δ_C_ 3.84/55.2 [6]. QuiNAc is a component of the LPS core oligosaccharide of brucellae [6] and its detection is consistent with the reactivity of these PS with the MAb of rough LPS specificity (see above). However, at the level where the H2/C2 cross-peak from QuiNAc was clearly visible, additional cross-peaks at δ_H_/δ_C_ 4.04/51.7 and 3.50/56.2 were present in the *B. suis* biovar 1 PS but absent in the biovar 2 PS. The former correlation is fully consistent with the chemical shifts for H4/C4 of a 3-substituted α-linked d-Rha*p*4NFo residue in the major *Z*-isomer also referred to as the s-*cis* conformation of the formyl group [20] and the latter with the minor *E*-isomer (s-*trans* conformation) of the amide substituent [21]. From ^1^H,^13^C-HSQC-^1^H,^1^H-TOCSY experiments [25], [26] with mixing times up to 200 ms, correlations from C4 atoms to H1 atoms were observed in the 2D NMR spectra. In particular, a correlation between δ_C_ 51.7 and δ_H_ 5.07 was present, i.e., between atoms from a structural element present only in the *B. suis* biovar 1 PS. Again, these results show that the extent of 3-substituted α-linked d-Rha*p*4NFo residues in the *B. suis* biovar 2 PS is lower than in the biovar 1 PS.

Integration of the methyl group resonance of the *N*-acetyl group of QuiNAc at δ_H_ 2.07 in the ^1^H NMR spectrum from the *B. suis* biovar 1 PS compared to protons in the anomeric region between δ_H_ 4.97 and 5.39 showed that the average number of sugars in the O-antigen polysaccharide was 50 to 60. The result for the *B. suis* biovar 2 PS was similar, which rules out the possibility that the above-described differences could be caused by a different degree of polymerization of the O-antigens. It was also noted that the anomeric protons at δ_H_ 5.26 and 5.10 showed correlations in the ^1^H,^13^C-HMBC spectrum to resonances at δ_C_ ∼74.3 which could be confirmed to be intraresidue correlations since these connectivities were also observed in the HSQC-TOCSY spectra; notably these sugar residues did not give any correlations to methyl groups at δ_H_ ∼1.21 and should originate from sugar residues other than d-Rha*p*4NFo in the polymer, such as sugars in the core region. We did not find any support for a significant amount of consecutively α-(1→3)-linked d-Rha*p*4NFo residues since the ^1^H chemical shifts of the anomeric protons should then reside at slightly lower chemical shifts than observed herein, like in the O-antigen polysaccharide from *Citrobacter gillenii* O9a,9b lipopolysaccharide [27].

The one-dimensional ^13^C NMR spectra of the *B. suis* biovar 1 and 2 PS were further analyzed in comparison with the spectra of *Y. enterocolitica* O:9 PS, and a few additional conspicuous differences were observed. In particular, the following resonances were present in the *B. suis* biovar 1 PS but absent in the biovar 2 PS: 18.0, 51.7, 102.4 and in particular 165.2 (Fig. 2d and 2e) and, noteworthy, they were also absent in the ^13^C NMR spectrum of *Y. enterocolitica* O:9 PS. However, in the latter PS, resonances of low intensity were observed at, inter alia, 102.5, 165.5 and 165.8 ppm (Fig. 2f). The ^13^C NMR chemical shift at 165.2 ppm in *B. suis* biovar 1 PS is fully consistent with a previous interpretation showing that an α-(1→3)-linked *N*-formylated perosamine residue is present in this polysaccharide [7] and confirms that the degree of α-(1→3)-linked d-Rha*p*4NFo residues is higher in the *B. suis* biovar 1 than in the biovar 2 O-antigen polysaccharides. Thus, the latter biovar may altogether lack the α-(1→3)-linkage in the O-antigen part of the PS.

To explore possible gene differences that could account for the O-antigen structural variation, the four known *Brucella* O-antigen glycosyltransferases were examined. To this end, the amino acid sequences of the *B. melitensis* 16M (biovar 1; highest proportion [21%] of α-(1→3)-linked d-Rha*p*4N) WboB (BMEI0997), WboA (BMEI0998), WbkE (BMEI1394) and WbkA (BMEI1404) were compared with the homologous genes in *B. suis* biovar 1 and 2 (no detectable α-(1→3)-linked d-Rha*p*4N). These analyses did not reveal any significant differences in any of these proteins. Similarly, no differences were found in the regions situated 300 bp upstream (putative promoter region) for any of the corresponding genes. A comparison of the amino acid sequences of other gycosyltransferases of *B. melitensis* 16M, *B. suis* biovar 1 and *B. suis* biovar 2 identified in data bases as presumably involved in LPS synthesis did not reveal significant differences but for *B. melitensis* BMEII 0728 and BMEII 1129 and their orthologues. BMEII 0728 carries a frame shift that results in the loss of 140 amino acids with regard to the orthologues (614 amino acids) in biovars 1 of *B. abortus* and *B. suis*. Since *B. melitensis* biovar 1 O-antigen shows about 21% of these linkages and *B. abortus* and *B. suis* show a similarly low proportion (about 2%) of α-(1→3)-linkages [7], these ORF are unlikely to play a role in the structural differences. BMEII 1129 is conserved in the genomes of biovars 1 of *B. melitensis*, *B. abortus* and *B. suis* but not in *B. suis* biovar 2, where it carries a frame shift. However, since BMEII 1129 mutant has been shown to maintain the O-antigen [6], either this glycosyltransferase is not related to the α-(1→3)-linkage or this linkage is not necessary for O-antigen polymerization in *B. melitensis*. Research is in progress to test these possibilities. Nonetheless, it seems that alternative and/or additional mechanisms of control are necessary to account for the different levels of α-(1→3)-linkages observed in the biovar 1 strains of *B. melitensis*, *B. abortus* and *B. suis*.

The proportions of α-(1→3)-linkages reported previously for the O-antigens of brucellae vary from 21% for *B. melitensis* biovar 1 strain 16M to 2% in *B. abortus* biovar 1 strain 1119-3, with intermediate values for *B. melitensis* biovar 3 (8%) and *B. suis* biovar 4 (13%) [7]. Therefore, the present work extends this range to include brucellae where this linkage is not detectable and could even be absent. Furthermore, during the last decade, *Brucella* has been identified as a pathogen of several marine mammals, and MAb reactivity patterns almost identical to that described here for *B. suis* biovar 2 have been reported for *B. ceti* strains isolated from some dolphin species [28]. Also, like *B. suis* biovar 2, several *Brucella* strains isolated from wild rodents in Australia do not react with MAb of C and M specificities [29]. Thus, it seems that the A-positive, C and M-negative epitopic structure is not infrequent outside the *B. abortus* and *B. melitensis* clusters and that it represents a new and extended *Brucella* serovar. The zoonotic nature of those dolphin and wild rodent strains is not known but considerable epidemiological evidence shows that *B. suis* biovar 2 displays little virulence for human beings. S-LPS is a major virulence factor of brucellae and it has been shown [30] that the O-antigen of virulent *B. suis* biovar 1 plays a critical role in selecting lipid rafts to enter into murine macrophages indicating a critical role at the port of entry. Since the O-antigen is the major surface structure, it is tempting to speculate that an absence of α-(1→3)-linkages may alter the interaction with human cells. Although this possibility cannot be ruled out, the fact is that the structural difference is small.

The results of this study show that the O-antigen of *B. suis* biovar 2 differs in a subtle but epitopically significant feature related to α-(1→3)-linkages in *Brucella* O-antigens. In this context, our results support the early interpretation that the α-(1→3)-linkage is partially responsible for the reactivity of those antibodies that recognize A and M dominant brucellae but not *Y. enterocolitica* O:9 [7]. Originally described as part of A [7], they are better defined as overlapping C (A = M and M>A) epitope(s) recognized by antibodies whose reactivity strictly requires the α-(1→3)-linkage in addition of a variable number of α-(1→2)-linked sugar residues. They are, therefore, different from those (the overlapping A and C/Y epitopes) attributable to α-(1→2)-linkages only. From a practical point of view, the C epitope(s) marks the only known serological difference between S brucellae and *Y*. *enterocolitica* O:9 but is undetectable in *B. suis* biovar 2. Swine are infected by *B. suis* biovar 2 in areas where infections by *Y*. *enterocolitica* O:9 also occur making necessary a differential diagnosis. The absence of antigenically detectable differences in the O-antigen of these two bacteria poses a very difficult challenge to the differentiation of these infections in standard LPS tests.

## Methods

### Strains, Growth Conditions and Polysaccharide Preparations

*B. suis* 1330 (biovar 1) and *B. suis* Thomsen (biovar 2; ATCC23445) are both defined serologically as A-dominant in biovar typing schemes [14]. *Y*. *enterocolitica* O:9 MY79 is an avirulent (plasmid-less) strain showing the expected cross-reactivity with S-brucellae [31]. The bacteria were grown and harvested as described previously [19] and S-LPS was extracted by the modified phenol-water method and purified by nuclease and proteinase K digestion [32]. The lipid A-free polysaccharide (PS) was obtained by hydrolysis of S-LPS with dilute aqueous acetic acid followed by ultracentrifugation and the purity of these fractions was demonstrated by quantitative immunoprecipitation [19] and with anti-core MAb (see Results).

### Enzyme-linked Immunosorbent Assay (ELISA)

ELISA was performed on polystyrene plates coated by incubation with the lipid A-free PS (see above) at 2.5 µg per ml in 10 mM phosphate buffered saline (pH 7.2) at 4°C overnight [15]. The relevant characteristics of the MAb used and appropriate references on their origin and production are summarized in Table 1.

### NMR Spectroscopy

1D and 2D NMR spectra of *B. suis* biovar 1 PS (10 mg in 0.5 mL D_2_O), biovar 2 PS (5 mg in 0.5 mL D_2_O) and *Y. enterocolitica* O:9-PS (9 mg in 0.5 mL D_2_O) were recorded at 47°C on a Bruker AVANCE III 700 MHz spectrometer equipped with a 5 mm TCI Z-Gradient CryoProbe. ^1^H chemical shifts were referenced to internal sodium 3-trimethylsilyl-(2,2,3,3-^2^H_4_)-propanoate (TSP, δ_H_ 0.00) and ^13^C chemical shifts were referenced to external dioxane in D_2_O (δ_C_ 67.40). Data processing was performed using vendor-supplied software. The assignments of the ^1^H and ^13^C resonances of the PS were obtained by analysis of 1D ^1^H and ^13^C NMR spectra together with 2D NMR spectra from multiplicity-edited^ 1^H,^13^C-HSQC experiments [33], ^1^H,^13^C-HMBC experiments [24] with a 45 ms delay for evolution of long-range couplings and ^1^H,^13^C-HSQC-^1^H,^1^H-TOCSY experiments [25], [26] with mixing times of 50, 100, 150 and 200 ms. A ^1^H,^1^H-NOESY experiment [34] with a mixing time of 100 ms was also performed for the *B. suis* biovar 1 PS.

### Sequence Analysis

Homologies of Brucella O-antigen glycosyltransferases *wboA, wboB, wbkA and wbkE* and upstream sequences and of the predicted proteins were studied using the NCBI (National Center for Biotechnology Information) and TIGR (The Institute for Genomic Research) sequences accessible through the *Bucella* Bioinformatics Portal (http://www.phidias.us/bbp/data/index.php) and BLASTN and BLASTP tools. For other Brucella LPS possible glycosyltranferases, the data available at the Carbohydrate Active Enzymes database (http://www.cazy.org/GlycosylTransferases.html ) were used.
